# Identification of a Phylogenetically Divergent Vanillate O-Demethylase from *Rhodococcus ruber* R1 Supporting Growth on *Meta*-Methoxylated Aromatic Acids

**DOI:** 10.3390/microorganisms11010078

**Published:** 2022-12-27

**Authors:** Raúl A. Donoso, Ricardo Corbinaud, Carla Gárate-Castro, Sandra Galaz, Danilo Pérez-Pantoja

**Affiliations:** 1 Programa Institucional de Fomento a la Investigación, Desarrollo e Innovación (PIDi), Universidad Tecnológica Metropolitana, Santiago 8940577, Chile; 2 Center of Applied Ecology and Sustainability (CAPES), Santiago 6513677, Chile

**Keywords:** plant-derived phenolics, rieske-type oxygenases, ring-hydroxylating oxygenases, actinobacteria, lignin valorization

## Abstract

Rieske-type two-component vanillate O-demethylases (VanODs) catalyze conversion of the lignin-derived monomer vanillate into protocatechuate in several bacterial species. Currently, VanODs have received attention because of the demand of effective lignin valorization technologies, since these enzymes own the potential to catalyze methoxy group demethylation of distinct lignin monomers. In this work, we identified a phylogenetically divergent VanOD from *Rhodococcus ruber* R1, only distantly related to previously described homologues and whose presence, along with a 3-hydroxybenzoate/gentisate pathway, correlated with the ability to grow on other meta-methoxylated aromatics, such as 3-methoxybenzoate and 5-methoxysalicylate. The complementation of catabolic abilities by heterologous expression in a host strain unable to grow on vanillate, and subsequent resting cell assays, suggest that the *vanAB* genes of R1 strain encode a proficient VanOD acting on different vanillate-like substrates; and also revealed that a methoxy group in the *meta* position and a carboxylic acid moiety in the aromatic ring are key for substrate recognition. Phylogenetic analysis of the oxygenase subunit of bacterial VanODs revealed three divergent groups constituted by homologues found in Proteobacteria (Type I), Actinobacteria (Type II), or Proteobacteria/Actinobacteria (Type III) in which the R1 VanOD is placed. These results suggest that VanOD from R1 strain, and its type III homologues, expand the range of methoxylated aromatics used as substrates by bacteria.

## 1. Introduction

The *Rhodococcus* genus is characterized by displaying a diverse range of metabolic capabilities, comprising degradation of short-chain, long-chain, and halogenated hydrocarbons; and numerous aromatic compounds, including substituted aromatics, heteroaromatics, and polycyclic aromatic hydrocarbons [1,2,3,4]. Accordingly, *Rhodococcus* spp. are considered as promising degraders of persistent contaminants, offering a multitude of novel enzymes able to perform challenging reactions [5,6,7].

Recently, we isolated a bacterial strain belonging to the *Rhodococcus* genus from a pulp mill wastewater treatment plant identified as *Rhodococcus ruber* strain R1, whose genome sequence consisted of one chromosome (~5.3 Mbp) and two plasmids (~179 and ~33 kbp), revealing an extensive catabolic potential [8]. Strain R1 has the ability to grow on various lignin-derived phenolic monomers, including *p*-coumarate and 4-hydroxybenzoate [8], which are typically catabolized via β-ketoadipate through the ring-cleavage intermediate protocatechuate (PCA) in *Rhodococcus* species [9,10]. Accordingly, we have currently confirmed that strain R1 is capable to grow on vanillate (VA), an additional lignin-derived product generated from the metabolism of ferulate and vanillin [10,11], as a sole carbon and energy source. The VA catabolism also proceeds via the PCA intermediate in several bacterial species, such as *Acinetobacter*, *Comamonas*, *Corynebacterium*, *Pseudomonas*, *Streptomyces,* and *Rhodococcus* as well, and is mediated by a two-component enzyme called vanillate O-demethylase (VanOD). This enzyme comprises a Rieske domain-containing oxygenase subunit encoded by *vanA*, and a reductase subunit that encompasses FMN, NADPH, and [2Fe-2S] cluster binding domains encoded by *vanB*, and providing electron equivalents to enable the enzymatic conversion [11,12,13,14,15,16,17,18,19]. It was shown that the VanOD encoded by *vanAB* genes from different bacteria were able to catalyze two types of reaction: methoxy group demethylation at the *meta* position of VA and analogs such as 3-methoxybenzoate (3-MB), veratrate, or syringate, with concomitant release of formaldehyde; or methyl group hydroxylation in *m*-toluate, 4-hydroxy-3-methylbenzoate, or 4-hydroxy-3,5-dimethylbenzoate; although, with the exception of veratrate and syringate, none of the analogs have been reported to support cell growth employing this enzyme [16,18,19,20,21]. Alternatively, it has been reported that distinct tetrahydrofolate (H_4_folate)-dependent O-demethylases, analogous to aromatic O-demethylases from anaerobic bacteria, are responsible for VA O-demethylation in *Sphingobium* sp. SYK-6 [22,23,24,25,26,27]. Remarkably, VanOD has gained increased interest since biocatalytic dealkylation of aryl methyl ethers have become attractive reactions for valorization of lignin-derived components towards fine chemicals and polymer precursors [28], highlighting the relevance of substrate-range studies for different VanOD enzymes.

**Figure 1:**
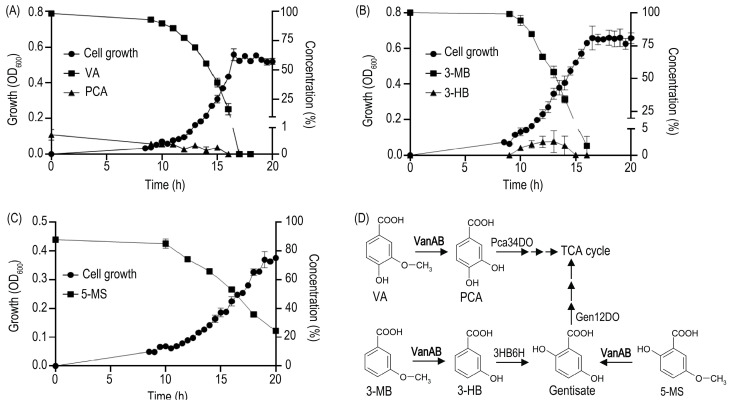
Growth curves of *Rhodococcus ruber* R1 on VA and analogs as sole carbon and energy sources, and their channeling into catabolic routes after O-demethylation. Growth of R1 strain on 5 mM (**A**) VA, (**B**) 3-MB, and (**C**) 5-MS as sole carbon and energy sources. Detection of (**A**) PCA and (**B**) 3-HB indicates O-demethylation activity in degradation of VA and 3-MB, respectively. In (**C**) 5-MS-grown cells, its O-demethylation product (gentisate) was not detected, suggesting rapid and efficient gentisate turnover in R1 strain. (**D**) Catabolic pathways for VA, 3-MB, and 5-MS predicted from inspection of *R. ruber* R1 genome. The first step in the VA degradation pathway is O-demethylation into the dihydroxylated intermediate protocatechuate (PCA), employing the *vanAB*-encoded enzyme (VanAB) as reported in *Rhodococcus jostii* RHA1 [11]. On the other hand, the O-demethylation of 3-MB could generate 3-HB, that is converted by action of a flavoprotein hydroxylase (3HB6H) into gentisate; which in turn, is the putative product of 5-MS O-demethylation. The route for 3-HB turnover through gentisate has been previously described in *R. jostii* RHA1 [38,39]. VanAB, Vanillate O-demethylase; Pca34DO, protocatechuate 3,4-dioxygenase; 3HB6H, 3-hydroxybenzoate 6-hydroxylase; Gen12DO, gentisate 1,2-dioxygenase; TCA, tricarboxylic acid. Optical density at 600 nm (closed circles); and the concentrations (%) of VA, 3-MB or 5-MS (closed squares), and PCA or 3-HB (closed triangles) are depicted in the figure. Concentrations are represented as percentages of the initial substrate concentration. Three biological replicates were performed for growth measurements. Error bars indicate SEM.

Surprisingly, the bacterium *Rhodococcus ruber* R1 only harbors VanAB proteins that are very distant from the well-known rhodococcal VanOD homologue described in *Rhodococcus jostii* RHA1 [11], showing only 36% amino acid identity for both subunits. Moreover, alternative tetrahydrofolate (H_4_folate)-dependent O-demethylases for VA, as those described in *Sphingobium* sp. SYK-6 [25], were not identified in its genome. On the other hand, PCA was transiently detected during growth of R1 strain in VA (Figure 1A), revealing that O-demethylation is occurring during its catabolism, and suggesting that a phylogenetically divergent VanOD encoded by putative *vanAB* genes (locus tag: E2561_01225 and E2561_01230) could be effectively responsible for VA catabolism in this bacterium. This interesting observation raises questions about possible differences among this divergent VanOD and the canonical one described in *R. jostii* RHA1 [11], its potential supporting role in the consumption of alternative substrates, and the distribution of close homologues in different *Rhodococcus* species.

## 2. Materials and Methods

### 2.1. Bacterial Strains, Plasmids, and Growth Conditions

Bacteria and plasmids used in this study are listed in Table 1. *Rhodococcus* species and *Cupriavidus pinatubonensis* JMP134 derivatives were grown at 30 °C in mineral salts medium previously reported [29], supplemented with 5 mM VA, PCA, 3-methoxybenzoate (3-MB), 5-methoxysalicylate (5-MS), gentisate, 3-hydroxybenzoate (3-HB), 3-methoxyphenylacetate, 3-hydroxyphenylacetate, 3-methoxysalicylate, 2,3-dihydroxybenzoate, syringate and homovanillate, as sole carbon and energy source. *Escherichia coli* Mach1 (Invitrogen, Carlsbad, CA, USA) was grown at 37 °C in Luria-Bertani (LB) medium. Growth was measured by optical density at 600 nm (OD_600_) using a spectrophotometer Spectroquant^®^ Prove (Merck, Darmstadt, Germany), or a Synergy HTX Multi-Mode plate reader (BioTek, Winooski, VT, USA). At least three biological replicates were performed for each growth measurement.

### 2.2. Construction of a Plasmid Expressing vanAB Genes and Growth Tests of Strain Derivatives

To obtain pBS1-*vanAB* plasmid (Table 1), which contain the *vanAB* genes under the control of an L-arabinose-inducible promoter, a restriction enzymes approach was used. In brief, PCR product comprising *vanAB* genes (locus tags: E2561_01225–E2561_01230), was obtained using oligos FW_vanAB_R1_EcoRI (5′-TGACGAATTCGAAGGAACGACATGACCGATC-3′) and RV_vanAB_R1_XbaI (5′-GTACTCTAGATGTATCCGATGACCAGGCC-3′) including underlined restriction sites for EcoRI and XbaI enzymes. The amplified DNA fragment was purified and double digested to be ligated into EcoRI/XbaI restriction sites of pBS1 [30], forming pBS1-*vanAB* plasmid, that was electroporated into *E. coli* Mach1. Transformed cells were selected in LB medium supplemented with gentamycin 30 µg mL^−1^; and selected transformants were checked by PCR for proper insertion of the *vanAB* genes. The full-length gene construct was again checked by Sanger sequencing for errors, and the pBS1-derived plasmid was transferred into strain JMP134 for phenotypic analysis. To evaluate growth proficiency, derivatives of JMP134 strain carrying *vanAB*-expressing plasmid were grown overnight on LB medium, and then inoculated at 0.2% in cultures containing 5 mM VA, PCA, 3-MB, 3-HB, 5-MS or gentisate as the sole carbon and energy source. For expression of *vanAB* genes driven by the heterologous *P_BAD_* promoter, these derivatives were exposed to 1 mM L-arabinose in addition to growth substrates. The cultures were incubated in a 96-well microplate (Thermo Fisher Scientific, Rochester, NY, USA) at 30 °C and the OD_600_ was recorded in a Synergy HTX Multi-Mode plate reader (BioTek, Winooski, VT, USA). 

### 2.3. Resting Cell Assays 

Resting cells of strain JMP134 derivatives were grown on 5 mM VA or 3-HB plus 1 mM arabinose where appropriate. These cells were washed twice with 1 volume of phosphate buffer (14 g/L Na_2_HPO_4_·12H_2_O, 2 g/L KH_2_PO_4_), 5X concentrated, and subsequently incubated with 1 mM of each compound to be assayed where appropriate. Samples were obtained at different times, filtered (0.22 µm), and stored at −20 °C.

### 2.4. Analytical Methods

The presence of VA, PCA, 3-MB, 3-HB, 5-MS, gentisate, syringate, 3-O-methylgallate, 3-methoxysalicylate, homovanillate, isovanillate, 3-methoxyphenylacetate, 2-methoxybenzoate, 4-methoxybenzoate, and guaiacol was determined by high performance liquid chromatography (HPLC) using cell-free supernatants. Samples were injected into a JASCO liquid chromatograph LC-4000 (JASCO, Okhaloma City, OK, USA) equipped with a Kromasil 100-3.5-C18 4.6 mm diameter column. A gradient HPLC method was used, which consists of a mobile phase composed of solvent A (solution containing formic acid 0.1% *v*/*v* in water) and solvent B (methanol), at a flow rate of 0.8 mL min^−1^. The initial mobile phase composition was maintained at 25% solvent B for 11 min, changed linearly to 55% (11–13 min) and finally it was kept at 55% solvent B for 7 min (13–20 min). The column effluent was monitored at 295 nm for VA, PCA, 3-MB, 3-HB, and 2-methoxybenzoate; 275 nm for syringate, 3-O-methylgallate, 3-methoxyphenylacetate, homovanillate and guaiacol; 320 nm for 5-MS, 3-methoxysalicylate and gentisate; and 260 nm for isovanillate and 4-methoxybenzoate. Retention times for VA, 3-MB, 3-HB, 2-methoxybenzoate, syringate, 3-O-methylgallate, 3-methoxyphenylacetate, homovanillate, guaiacol, 5-MS, 3-methoxysalicylate, isovanillate, 4-methoxybenzoate, PCA, and gentisate were 9.3, 18.7, 10.9, 15.9, 10.5, 5.0, 17.7, 10.1, 15.8, 18.1, 16.6, 10.2, 18.1, 4.4, and 7.2 min, respectively.

### 2.5. Bioinformatic Tools

The *vanAB* gene sequences from different bacterial species were retrieved from non-redundant protein sequences database of GenBank (https://blast.ncbi.nlm.nih.gov/Blast.cgi, accessed on 8 September 2022) [31]. Only proteins displaying at least 60% amino acid identity with previously described VanA from *Pseudomonas* sp. HR199 [14], *Rhodococcus jostii* RHA1 [11], and *Rhodococcus ruber* R1 (this study) were recruited for analysis using BLAST software [32].

Evolutionary relationships were inferred by IQ-TREE web server tools (http://iqtree.cibiv.univie.ac.at/, accessed on 8 September 2022) proposed for estimate maximum-likelihood phylogenies [33] employing ModelFinder as model-selection method [34] and UFBoot2 for ultrafast bootstrap approximation [35] with the -m TEST, -bb 1000, and -alrt 1000 settings. Sequence alignments for phylogenetic reconstruction were calculated using MAFFT software online server (https://mafft.cbrc.jp/alignment/server/, accessed on 8 September 2022) employing Auto strategy (FFT-NS-1, FFT-NS-2, FFT-NS-i or L-INS-i; depending on data size) [36]. Edition and visualization of dendrograms was performed by the Interactive Tree of Life (iTOL) online tool (https://itol.embl.de/, accessed on 8 September 2022) [37].

### 2.6. Chemicals

VA, PCA, 3-MB, 3-HB, 5-MS, gentisate, 2,3-dihydroxybenzoate, syringate, 3-O-methylgallate, 3-methoxysalicylate, homovanillate, isovanillate, 3-hydroxyphenylacetate, 3-methoxyphenylacetate, 2-methoxybenzoate, 4-methoxybenzoate, and guaiacol were purchased from Sigma-Aldrich (Steinheim, Germany). L(+)-arabinose was purchased from Merck (Darmstadt, Germany).

## 3. Results and Discussion

### 3.1. Heterologous Expression and Resting Cell Assays Suggest a Key Role of VanOD from Rhodococcus ruber R1 in meta-Methoxylated Aromatic Acids Degradation

Our original observation about the ability of *R. ruber* R1 to grow on VA as a sole carbon and energy source (Figure 1A), in the absence of a canonical VanOD as the one described in *R. jostii* RHA1 [11], prompted us to analyze its growth profile on other *meta*-methoxylated aromatic substrates, such as 3-methoxybenzoate (3-MB) and 5-methoxysalicylate (5-MS). Interestingly, 3-MB and 5-MS also supported cell proliferation (see Figure 1B,C for a detailed growth curve of R1 cells), suggesting O-demethylation of these substrates into 3-hydroxybenzoate (3-HB) and gentisate respectively, as depicted in Figure 1D. For 3-MB consumption, the O-demethylation activity was additionally suggested by the transient accumulation of 3-HB (Figure 1B). In the case of 5-MS growth, the absence of gentisate in the supernatant of R1 cell cultures would be correlated with a lower rate of substrate consumption, in comparison to VA and 3-MB consumption curves, as shown by Figure 1A–C; avoiding the accumulation of intermediates. Further catabolism of 3-HB and gentisate is correlated with the presence of genes encoding 3-hydroxybenzoate 6-hydroxylase (locus tag: E2561_07550) and gentisate 1,2-dioxygenase (locus tag: E2561_07565) enzymes, comprising the catabolic route for 3-HB via gentisate in strain R1, which are closely related to the enzymes described for *R. jostii* RHA1 [38,39]. The presence of O-demethylation activities for VA, 3MB, and 5-MS in *R. ruber* R1 raise the possibility that VanOD encoded in the genome of this strain would be responsible for all of them. 

**Figure 2:**
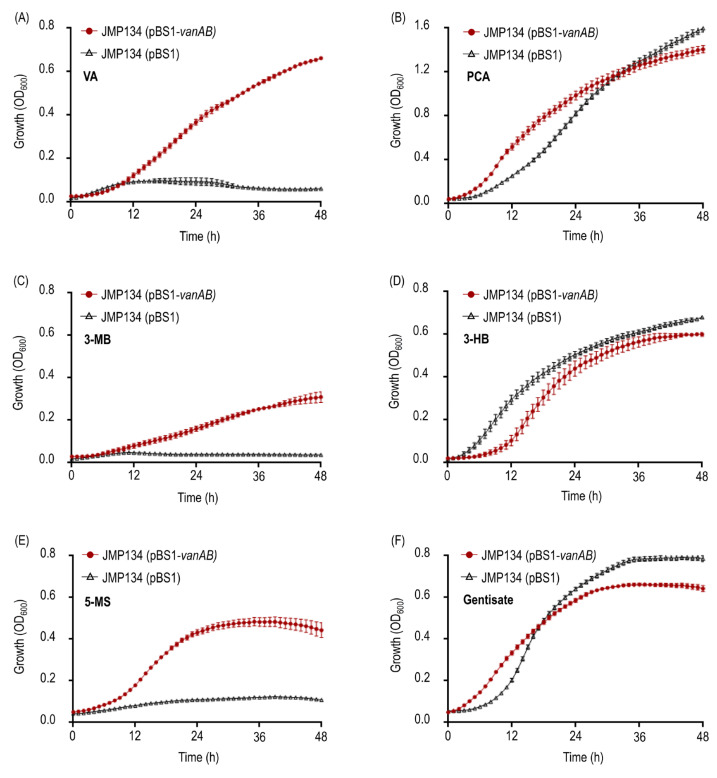
Growth on *meta*-methoxylated aromatic acids, VA, 3-MB, and 5-CS, and their O-demethylation products of *Cupriavidus pinatubonensis* JMP134 expressing *vanAB* genes of *R. ruber* R1. Growth of *C. pinatubonensis* JMP134 expressing *vanAB* genes driven by a heterologous *P_BAD_* promoter on 5 mM (**A**) VA, (**B**) PCA, (**C**) 3-MB, (**D**) 3-HB, (**E**) 5-MS, or (**F**) gentisate as sole carbon and energy sources was assayed in the presence of 1 mM L-arabinose as inducer. Three biological replicates were performed for growth measurements. Error bars indicate the SEM.

**Figure 3:**
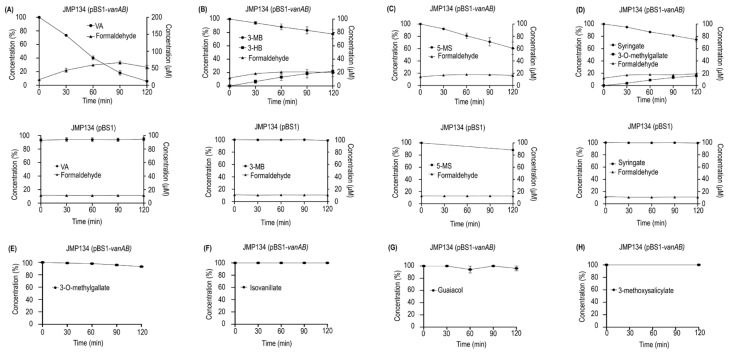
Resting cell assays of *Cupriavidus pinatubonensis* JMP134 harboring *vanAB* genes in the presence of compounds structurally related to VA. Cells of *C. pinatubonensis* JMP134 expressing *vanAB* genes were grown on 5 mM VA plus 1 mM L-arabinose as inducer, washed, and subsequently exposed to 1 mM (**A**) VA, (**B**) 3-MB, (**C**) 5-MS, (**D**) syringate, (**E**) 3-O-methylgallate, (**F**) isovanillate, (**G**) guaiacol, and (**H**) 3-methoxysalicylate. Cells of *C. pinatubonensis* JMP134 lacking *vanAB* genes were grown on 5 mM 3HB plus 1 mM L-arabinose and treated as indicated previously for comparison. Three biological replicates were performed for substrate consumption measurements. Error bars indicate SEM.

In order to gain comprehension about the whole function of the divergent VanOD from *R. ruber* R1, a plasmid construct containing the *vanAB* genes of this strain was introduced into *C. pinatubonensis* JMP134, a well-known aromatics-degrader bacterium unable to grow on VA, 3-MB, and 5-MS, but that harbors PCA, 3-HB, and gentisate degradation routes (Figure 2B,D,F) [40], allowing complementation of the catabolic abilities. The expression of the *vanAB* genes was controlled by the L-arabinose-inducible *P_BAD_* promoter, which was chosen since L-arabinose is non-toxic and is not a carbon source for *C. pinatubonensis* JMP134, permitting reliable growth tests in this strain [30,41]. Remarkably, the presence of *vanAB* genes was sufficient to allow L-arabinose-depending growth on VA, 3-MB, and 5-MS of JMP134 strain (Figure 2A,C,E), strongly suggesting that VanOD of R1 strain has O-demethylation activity toward the three *meta*-methoxylated aromatic substrates. It should be noted that, in the absence of L-arabinose as an inducer, no growth was observed (data not shown), and that the presence of the empty pBS1 vector has no effect on cell proliferation of JMP134 strain on these substrates (Figure 2A,C,E). Moreover, resting cell assays of JMP134 (pBS1-*vanAB*) cells previously grown on VA showed a sharp decrease in the concentration of VA, and a slower consumption rate for 3-MB and 5-MS, also detecting the occurrence of 3-HB in 3-MB-incubated cells (Figure 3A–C); which provides further support for VA/3-MB/5-MS O-demethylase activity encoded by R1 *vanAB* genes. This inference was additionally supported by detecting a small accumulation of formaldehyde in parallel to substrates consumption (Figure 3A–C), which is the by-product of O-demethylation by VanOD [26]. 

The presence of functional groups in the potential substrates of the divergent VanOD of R1 strain was the next interesting issue to be determined. Nishimura et al. [19] reported that a carboxylic acid on the benzene ring in conjunction with a hydroxyl group in *para*-orientation, as occurs in VA or syringate molecules, is required for efficient methoxy oxidation in *meta*-position of the VanAB substrates in *Streptomyces* sp. NL15-2K, which is homologous to VanAB from RHA1 (70% aa identity for oxygenase subunit). Recently, the properties of VanOD from *Pseudomonas* sp. HR199 were extensively examined, confirming that the presence of a carboxylic acid moiety is essential, and that catalysis occurs selectively at the *meta*-position relative to the –COOH group in the aromatic ring, although exposing specific differences in substrate recognition in comparison to VanAB from *Streptomyces* sp. NL15-2K [19,28]. To confirm that previous observations also apply to VanOD from *R. ruber* R1, resting cell assays considering additional potential substrates were performed in *C. pinatubonensis* JMP134 carrying the plasmid that contains the *vanAB* genes from R1 strain. The doubly *meta*-methoxylated syringate that carries a –OH group in the *para*-position relative to the carboxylic acid was a proper substrate for VanOD of the R1 strain; being 3-O-methylgallate, the partially demethoxylated analog, identified as the only conversion product of its catalysis (Figure 3D). Meanwhile, 3-O-methylgallate apparently was not recognized as a substrate by the VanOD of R1 strain (Figure 3E), similar to what was described for the VanAB from HR199 strain [28], but unlike VanAB of NL15-2K strain which is able to generate a mixture of 3-O-methylgallate and gallate, the fully demethoxylated analog, in the presence of syringate [19]. These results were supported by introduction of *vanAB* genes of R1 strain into *Pseudomonas putida* KT2440, which is unable to grow on syringate or 3-O-methylgallate but contains a functional gallate degradation pathway [42,43], being the product of two consecutive O-demethylations over syringate comprising 3-O-methylgallate as intermediate, as mentioned before. The *P. putida* KT2440 (pBS1-*vanAB*) strain was unable to grow on syringate as a sole carbon and energy source (data not shown), suggesting that inefficient O-demethylation of 3-O-methylgallate by VanOD from R1 could be the reason for this phenotype.

Additional compounds including differences in the key positions of functional groups for the recognition of substrates by this enzyme, such as 2-methoxybenzoate and 4-methoxybenzoate (methoxy group in *ortho*- or *para*-position in relation to –COOH group), isovanillate (methoxy group in *para*-position in relation to –COOH group with an adjacent –OH group in *meta*-position), homovanillate (VA analog with a –CH_2_COOH replacing –COOH group), 3-methoxyphenylacetate (3 MB analog with a –CH_2_COOH replacing –COOH group), 3-methoxysalicylate (methoxy group in *meta*-position in relation to –COOH group with an adjacent –OH group in *ortho*-position), and guaiacol (methoxy group with an adjacent –OH group but lacking –COOH group) were not degraded by resting cells of *C. pinatubonensis* JMP134 carrying the *vanAB* genes of R1 strain (see Figure 3F–H, for isovanillate, guaiacol, and 3-methoxysalicylate as representative examples).

In summary, the results of growth tests and resting cell assays suggest that divergent VanOD from strain R1 not only recognizes VA, but also 3-MB, 5-MS, and syringate as proper substrates to a lesser extent (Figure 3A–D). According to this, *vanAB* genes could be key not only on VA degradation, but also on the potential catabolism of 3-MB and 5-MS in additional *Rhodococcus* species that carry this divergent version of VanOD.

### 3.2. Rhodococcus Strains Carrying VanAB Homologues Closely Related to VanOD of R. ruber R1 Strain Are Able to Grow on VA, 3-MB, and 5-MS as a Sole Carbon and Energy Sources

**Figure 4:**
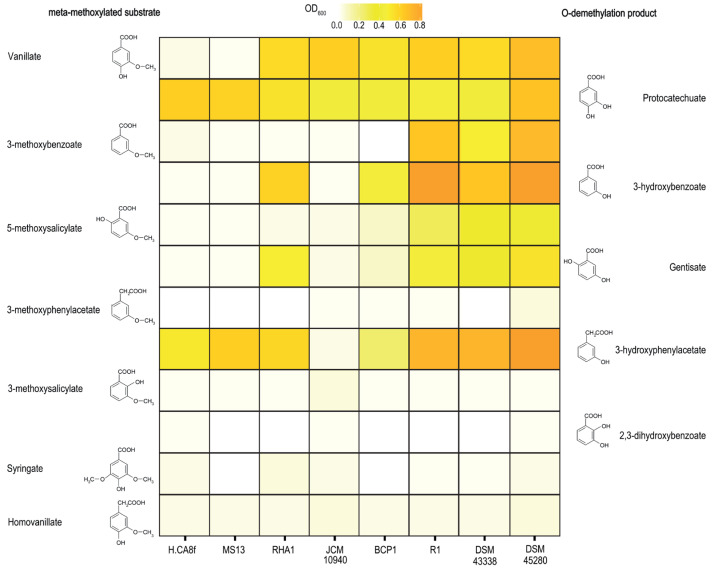
Growth on *meta*-methoxylated aromatic acids and their O-demethylated products of different *Rhodococcus* species. Strains belonging to *Rhodococcus* genus were grown in mineral salt medium with 5 mM of several *meta*-methoxylated aromatic acids (methoxylated substrates; left side) and its O-demethylated products (right side) as sole carbon and energy sources. Shading indicates optical density (OD) at 600 nm after 40 h (average of three biological replicates). It is worth mentioning that O-demethylated products related to syringate (3-O-methylgallate and gallate) and homovanillate (homoprotocatechuate) were rapidly oxidized in solution yielding an intense dark brown color on the medium, precluding determination of optical density, and consequently were excluded of the study.

To explore the phenotypic differences of selected *Rhodococcus* species carrying divergent VanOD related to VanAB from *R. jostii* RHA1 strain or VanAB from *R. ruber* R1 strain, we analyzed their growth profile on several *meta*-methoxylated substrates structurally related to VA, such as 3-MB, 5-MS, syringate, 3-methoxysalicylate, 3-methoxyphenylacetate, and homovanillate, in addition to some of the putative products of O-demethylation such as PCA, 3-HB, gentisate, 2,3-dihydroxybenzoate and 3-hydroxyphenylacetate (Figure 4).

Results showed that *Rhodococcus* strains harboring *vanAB*-like genes similar to *R. ruber* R1 as *R. ruber* DSM 43338^T^, *R. ruber* Chol-4 and *R. pyridinivorans* JCM 10940^T^ [8,44,45], and *vanAB* genes comparable to *R. jostii* RHA1 as *R. aetherivorans* BCP1 [46,47] were able to grow on VA and its O-demethylation product, PCA (Figure 4), suggesting that all these strains contain proficient VanOD-encoded genes and a functional PCA pathway (Figure 1D). We also included in our growth profile assays marine-isolated *Rhodococcus* strains MS13 and H-CA8f as control [48,49], since they apparently do not harbor VanOD-encoded genes, even though they carry the classical PCA pathway [50], as revealed by BLAST searches and confirmed by growth on PCA as a sole carbon and energy source (Figure 4). Accordingly, both *Rhodococcus* strains of marine origin were unable to grow on VA (Figure 4), confirming the previous bioinformatic survey that revealed the absence of VanOD encoded genes, and suggesting that VA degradation activity could be linked to *Rhodococcus* species found mainly in soil or freshwater environments, probably correlated to lignin depolymerization [51,52,53].

Remarkably, *R. ruber* strains DSM 43338^T^ and Chol-4 that harbor *vanAB* genes close related to R1 homologues were also able to use 3-MB and 5-MS, and its putative demethylation products, 3-HB and gentisate, respectively, as sole carbon and energy sources (Figure 4), suggesting that their VanOD enzymes are able to act on both *meta*-methoxylated substrates, and that they harbor the corresponding putative downstream pathways (Figure 1D). Notably, *R. pyridinivorans* JCM 10940^T^ containing R1-like *vanAB* genes was unable to grow on 3-MB and 5-MS, and was also unable to grow on 3-HB and gentisate (Figure 4), which is in accordance with the absence of genes encoding 3-HB 6-hydroxylase and gentisate 1,2-dioxygenase enzymes. This suggests that lack of a functional 3-HB/gentisate pathway might impair its growth on such *meta*-methoxylated substrates, regardless of the presence of a proficient VanOD. Conversely, despite strains RHA1 and BCP1 harbor RHA1-like *vanAB* genes and contain a functional 3-HB/gentisate pathway, both were unable to grow on 3-MB and 5-MS (Figure 4), suggesting that the VanOD harbored by these *Rhodococcus* strains does not support the O-demethylation activities toward these *meta*-methoxylated substrates. These results could indicate that the *in vivo* range of substrate acceptance for R1-like and RHA1-like VanOD enzymes is not the same. Finally, all *Rhodococcus* strains tested were unable to use the remaining VA analogs assayed as sole carbon and energy sources, including those where –COOH group is replaced by –CH2COOH as 3-methoxyphenylacetate or homovanillate (Figure 4).

### 3.3. Two-Component Rieske-Type VanOD of Rhodococcus Species Are Allocated in Two Divergent Phylogenetic Clades

**Figure 5:**
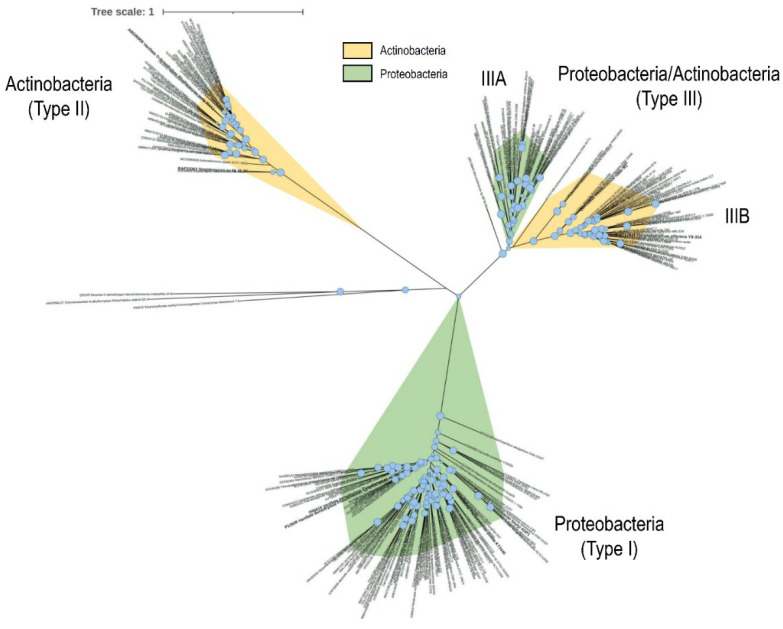
Evolutionary relationships among VanA homologues from bacteria. Maximum likelihood topology provided by IQ-TREE software [33] based on sequence alignments calculated using MAFFT software [36] is shown with SH-like approximate likelihood ratio support values (*n* = 1000) given at each node (values >70% are shown). Light orange, *Actinobacteria phyla*; green, *Proteobacteria phyla*.

The existence of at least two distinct VanOD types in *Rhodococcus* species prompted us to evaluate the distribution of each kind in this genus and other actinobacterial and proteobacterial genomes. For that purpose, we chose as gene marker the VanA product, coding the oxygenase component of the enzyme, from *R. jostii* RHA1 [11], *R. ruber* R1, and also *Pseudomonas* sp. HR199 as *bona fide* representative of proteobacterial VanOD [14]. Then, we conducted a search in the non-redundant protein sequences database of GenBank as of September 2022, selecting VanA from bacterial species displaying at least 60% amino acid identity in order to establish phylogenetic relationships. As a result of a high number of redundant VanA sequences, we selected one representative VanA homologue belonging to each genus identified. The resulting VanA phylogenetic tree showed three clearly divergent groups, in which a precise partition was perceived between a proteobacterial clade (called type I), including the well-known VanA homologues from *Acinetobacter baylyi* ADP1, *Pseudomonas* sp. HR199, and *Comamonas testosteroni* BR6020 [14,16,18]; and an actinobacterial clade (type II), including the aforementioned VanA homologues from *R. jostii* RHA1 and *Streptomyces* sp. NL15-2K [11,19] (Figure 5). Interestingly, a distinct third clade was detected (type III), internally partitioned in two subclades including homologues from Proteobacteria (type IIIA) and Actinobacteria (type IIIB) (Figure 5). The last one included VanA from *R. ruber* R1, revealing that the VanOD reported in this work is the first member of this clade whose functionality and substrate range is analyzed in detail. It should be noted that a closer inspection of each clade reveals a predominance of β- and γ-proteobacterial VanA homologues among members of the type I clade, meanwhile only homologues from Actinobacteria representatives were found in type II (data not shown). Conversely, a prevalence of VanA homologues from α-proteobacteria subclass representatives (IIIA) in conjunction with Actinobacteria (IIIB) were observed in type III (data not shown). 

**Figure 6:**
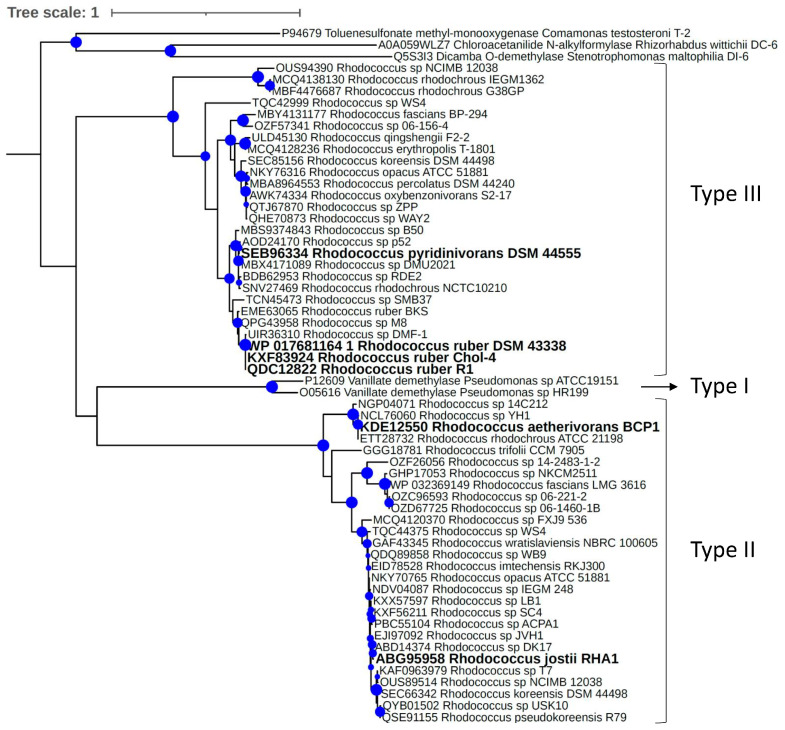
Evolutionary relationships among VanA homologues from *Rhodococcus* species. Maximum likelihood topology provided by IQ-TREE software [33] based on sequence alignments calculated using MAFFT software [36] with SH-like approximate likelihood ratio support values (*n* = 1000) given at each node (values >70% are shown). VanA homologues of *Pseudomonas* sp. ATCC 19151 and *Pseudomonas* sp. HR199 were included as representatives of the proteobacterial type I VanOD. Oxygenase components of Rieske-type 4-Toluene sulfonate methyl-monooxygenase from *Comamonas testosteroni* T-2, Chloroacetanilide N-alkylformylase from *Rhizorhabdus wittichii* DC-6, and Dicamba O-demethylase from *Stenotrophomonas maltophilia* DI-6 were used as outgroup. Sequences highlighted in bold belong to strains tested by their ability to grow in *meta*-methoxylated aromatic acids as a sole carbon and energy source.

In order to gain a deeper understanding of the phylogenetic relationships between VanOD enzymes from *Rhodococcus* species, an additional phylogenetic tree was constructed including VanA homologues from a broader range of *Rhodococcus* species representatives (Figure 6). Similar to what was previously observed, it was shown that VanA from *Rhodococcus* species are grouped either in conjunction with VanA from RHA1 strain (type II clade) or VanA from R1 strain (type III clade). The number of VanA homologues from *Rhodococcus* species grouped in each clade was roughly similar, suggesting that both types of VanOD are numerically relevant in this actinobacterial genus. No VanA homologue of the *Rhodococcus* species considered in this study was located out of these clades. 

## 4. Conclusions

Given the current interest in O-demethylation reactions for lignin conversion into renewable chemicals [54], this study aimed to shed light on *Rhodococcus* enzymes acting on *meta*-methoxylated aromatic compounds such as VA, one of the most prominent lignin-derived phenolics. This work revealed that *Rhodococcus* genus harbors at least two divergent types of VanOD-encoding genes represented by *vanAB* from *Rhodococcus jostii* RHA1 (Type II) and *vanAB* from *Rhodococcus ruber* R1 (Type III). Most interestingly, the VanOD from R1 strain is responsible for catabolism of additional *meta*-methoxylated phenolics such as 3-MB and 5-MS, as inferred from growth tests and resting cell assays of a heterologous strain expressing R1 *vanAB* genes, and from the substrate utilization pattern of *Rhodococcus* strains harboring close homologues of this enzyme. This expanded substrate specificity would be advantageous for metabolic engineering endeavors focused on bioconversion process toward renewable chemicals based on microbial demethylation of lignin monomers.

**Table 1:** Bacterial strains and plasmids used in this study.

Strain or Plasmid	Relevant Phenotype and/or Genotype	Reference or Source
***Rhodococcus* strains**		
*R. aetherivorans* BCP1	VA^+^, 3-MB^-^, 5-MS^-^	[47]
*R. jostii* RHA1	VA^+^, 3-MB^-^, 5-MS^-^	[46]
*R. ruber* R1	VA^+^, 3-MB^+^, 5-MS^+^	[8]
*R. ruber* Chol-4	VA^+^, 3-MB^+^, 5-MS^+^	[45]
*R. ruber* DSM 43338 ^T^	VA^+^, 3-MB^+^, 5-MS^+^	DSMZ ^a^
*R. pyridinivorans* JCM 10940 ^T^	VA^+^, 3-MB^-^, 5-MS^-^	[44]
*Rhodococcus* sp. H-CA8f	VA^-^, 3-MB^−^, 5-MS^-^	[48]
*Rhodococcus* sp. MS13	VA^-^, 3-MB^-^, 5-MS^-^	[49]
**Other strains**		
*E. coli* Mach1	∆recA1398 endA1 tonA Φ80∆lacM15 ∆lacX74 hsdR (rK^-^ mK^+^)	Invitrogen, Carlsbad, CA, USA
*C. pinatubonensis* JMP134	PCA^+^, Gentisate^+^, 3-HB^+^, VA^-^, 3-MB^-^, 5-MS^-^	[40]
**Plasmids**		
pBS1	Broad host range vector, *araC*-*P_BAD_*, Gm^R^	[55]
pBS1-*vanAB*	pBS1 derivative expressing *vanAB* genes, Gm^R^	This study

^a^ DSMZ, Deutsche Sammlung von Mikroorganismen und Zellkulturen GmbH (Braunschweig, Germany). ^T^: Type strain.

## Data Availability

The data supporting the conclusions of this work are included within the manuscript and there were no large datasets generated or analyzed during the current study.
